# Accuracy of Dynamic Computer-Assisted Surgery for Pterygoid Implant Placement in Fully and Partially Edentulous Maxillae: A Retrospective Comparative Study

**DOI:** 10.3390/medicina62091710

**Published:** 2026-09-06

**Authors:** Luka Plivelić, Ivica Dubravica, Ivan Zajc, Vlatka Debeljak, Ana Zulijani

**Affiliations:** 1 Faculty of Dental Medicine and Health of Osijek, Josip Juraj Strossmayer University of Osijek, 31000 Osijek, Croatia; luka.plivelic5@gmail.com; 2 Polyclinic Dubravica, 22211 Vodice, Croatia; drdubravica@gmail.com; 3 Department of Oral Surgery, School of Dental Medicine, University of Zagreb, Gundulićeva 5, 10000 Zagreb, Croatia; zajc@sfzg.unizg.hr; 4 Department of Maxillofacial and Oral Surgery, University Hospital Dubrava, Av. Gojka Šuška 6, 10000 Zagreb, Croatia; 5 Department of Dental Medicine, Faculty of Dental Medicine and Health of Osijek, Josip Juraj Strossmayer University of Osijek, Crkvena 21, 31000 Osijek, Croatia; azulijani@fdmz.hr; 6 Department of Prosthodontics, Faculty of Dental Medicine Rijeka, University of Rijeka, Krešimirova 40, 51000 Rijeka, Croatia; 7 Clinical Hospital Center Rijeka, Krešimirova 40, 51000 Rijeka, Croatia

**Keywords:** accuracy, dynamic computer-assisted implant surgery system, markerless tracing registration pterygoid implant, radiographic marker registration

## Abstract

*Background and Objectives:* The rehabilitation of the atrophic posterior maxilla with pterygoid implant presents a significant challenge due to complex regional anatomy and limited visual access. This study aimed to compare the accuracy of a dynamic computer-assisted implant surgery system (dCAIS) for pterygoid implant placement in fully and partially edentulous maxillae using radiographic marker registration (RMR) and markerless tracing registration (MTR), respectively. *Materials and Methods:* Forty pterygoid implants were retrospectively evaluated in 40 patients, divided into two groups: fully edentulous (n = 20) and partially edentulous (n = 20). Implant placement was performed using a dynamic navigation system (dCAIS), utilizing either bone-anchored mini-screws or tooth-surface tracing as reference points. Accuracy was assessed by superimposing preoperative plans with postoperative cone beam computer tomography (CBCT) scans. Coronal, apical, and angular deviations were measured and analyzed using the Data Science Workbench (version 14). The level of statistical significance was set at α = 0.05 (two-tailed). *Results:* The mean deviations for fully and partially edentulous maxillae measured 1.24 ± 0.27 mm and 1.40 ± 0.27 mm at the coronal level (*p* = 0.065), 1.30 ± 0.49 mm and 1.30 ± 0.54 at the apical level (*p* = 0.995), and 0.72 ± 0.38° and 0.82 ± 0.43° in angular deviation (*p* = 0.560), respectively. No statistically significant differences were found between the two groups. Notably, mean angular deviation was remarkably low (<1°) in both groups. *Conclusions:* No statistically significant between-group differences in accuracy were detected in coronal, apical, and angular deviations between the markerless tracing registration method in partially edentulous patients and the bone-anchored radiographic marker registration method in fully edentulous patients.

## 1. Introduction

The rehabilitation of the atrophic posterior maxilla represents a significant challenge in implant dentistry, primarily due to the marked reduction in available bone height and width resulting from the combined impact of alveolar bone resorption and pneumatization of the maxillary sinus [1,2,3,4]. In addition, the bone quality in this region is often poor, typically categorized as type III or IV, which can compromise primary implant stability and long-term osseointegration [4,5,6].

To address these limitations, various treatment approaches have been proposed, such as sinus floor elevation, guided bone regeneration, and the use of short or zygomatic implants [7,8,9,10,11]. However, these techniques are often associated with increased surgical morbidity, higher costs, and extended healing periods prior to final prosthetic loading. Additionally, these procedures may require multiple surgical interventions, potentially increasing the risk of complications and patient discomfort [11,12,13,14,15]. Ultimately, establishing standardized clinical indications and outcome assessment criteria for borderline cases of severe maxillary atrophy is essential for optimizing patient-specific treatment planning [11].

In recent years, alternative treatment strategies aimed at minimizing surgical invasiveness and reducing overall treatment time have gained increasing attention. Among these, the use of pterygoid implants has emerged as a promising solution for the rehabilitation of the atrophic posterior maxilla. Pterygoid implants, first introduced by Tulasne et al., were designed to bypass the maxillary sinus by anchoring in dense cortical bone of the pterygomaxillary region [16]. This anchorage involves the maxillary tuberosity, the pyramidal process of the palatine bone, and the pterygoid process of the sphenoid bone, providing enhanced primary stability in cases of posterior maxillary atrophy, enabling immediate loading and eliminating distal cantilevers [17,18,19].

Despite their advantages and high success rates of approximately 94.87%, the placement of pterygoid implants is technically demanding due to the complex anatomy of the pterygomaxillary region and limited visual access [20]. The importance of accurate implant positioning is particularly pronounced for this treatment modality because of the long trajectory through a confined anatomical corridor, where the proximity of relevant anatomical structures increases the potential consequences of trajectory deviations. The complications reported in the literature include bleeding and implant displacement of implants into the pterygoid or infratemporal fossa [21,22,23].

To improve placement accuracy and safety, computer-aided implant surgery (CAIS) has been introduced into clinical practice. CAIS can reduce deviations between virtual planning and actual implant placement, facilitating a prosthetically driven workflow based on 3D cone beam computed tomography (CBCT) data and specialized software. Furthermore, CAIS may enhance surgical predictability and patient safety by facilitating accurate implant positioning and reducing the risk of injury to vital anatomical structures [24,25,26,27]. In this evolving digital landscape, artificial intelligence (AI) and machine learning models are further transforming workflows in implant dentistry by supporting automated 3D planning, optimizing intraoperative navigation, and potentially facilitating prediction of treatment outcomes [28,29].

Two CAIS approaches have been developed: static guidance (static computer-aided implant surgery, sCAIS) and dynamic navigation (dynamic computer-aided implant surgery, dCAIS) [27,30,31]. Unlike sCAIS, where the use of static surgical templates may restrict irrigation and limit surgical visibility, dCAIS utilizes optical tracking to provide real-time feedback of the position of the drill relative to the preoperative plan, allowing greater intraoperative flexibility and potentially facilitating irrigation and cooling [27,32].

The registration process is a critical factor influencing navigation accuracy between the planned and actual implant position in the dCAIS system. Two commonly used registration workflows are radiographic marked registration (RMR) and markerless tracing registration (MTR) [33]. The RMR method requires radiographic markers incorporated into an intraoral template, clip, or bone-anchored mini-screws to be present during both the CBCT scan and the surgical procedure, serving as fixed fiducial reference points for the optical tracker. A potential advantage of RMR is the use of clearly identifiable and reproducible fiducial markers that can provide stable registration references when securely positioned. However, registration accuracy may be influenced by the number and geometric distribution of these fiducial markers. A wide and bilateral distribution may provide a more favorable geometric configuration than a localized or unilateral arrangement, particularly when the reference points are distant from the surgical site. Furthermore, any intraoperative displacement of the template or clip may compromise navigation accuracy. In contrast, the MTR method leverages the patient’s anatomy, typically the edges or cusps of the remaining teeth, by “tracing” these structures with an optical probe to synchronize the physical patient with the digital dataset. Although MTR avoids potential displacement of the registration template and simplifies the clinical workflow, its accuracy may still be influenced by the availability, stability, and geometric distribution of suitable residual anatomical landmarks [24,27,34,35,36,37].

Although dCAIS has been investigated, to our knowledge, limited clinical evidence is available regarding the accuracy of dCAIS for pterygoid implant placement in fully and partially edentulous maxillae. The aim of this exploratory retrospective comparative study was to evaluate and compare the accuracy of pterygoid implant placement in fully and partially edentulous maxillae using two clinically distinct workflows. Radiographic marked registration (RMR) was used in fully edentulous maxillae, whereas markerless tracing registration (MTR) was used in partially edentulous maxillae.

## 2. Materials and Methods

### 2.1. Study Design and Ethical Considerations

The study was designed as a retrospective comparative study based on the cone beam computed tomography (CBCT) scans of patients rehabilitated with pterygoid implants using a dynamic navigation system (Navident, ClaroNav Technology, Toronto, ON, Canada) in a private practice, Polyclinic Dubravica (Vodice, Croatia) between January 2024 and February 2025. Data collection was performed between April and June 2025. All patients who met the predefined inclusion and exclusion criteria were consecutively included. One pterygoid implant per patient was placed and analyzed, resulting in a total of 40 pterygoid implants in 40 patients. All implants placed were PteryFit from the Noris Medical system (Noris Medical, Nesher, Israel). The sample size was determined by the number of consecutive eligible patients during the predefined study period. Ethical approval was obtained from the Institutional Ethics Committee of Polyclinic Dubravica (Ref. No: 145/25). The study was conducted in accordance with the Declaration of Helsinki of 1975, as revised in 2013, and written informed consent was obtained from all patients.

### 2.2. Patient Selection

Forty patients (19 men and 21 women) who underwent pterygoid implant placement using the Navident system were included based on predefined inclusion and exclusion criteria. The inclusion criteria were as follows: (1) fully edentulous maxilla, (2) partially edentulous maxilla (with a minimum of four stable teeth and at least 2 teeth per quadrant), (3) American Society of Anesthesiologists (ASA) physical status I or II, and (4) availability of both preoperative and postoperative CBCT scans of sufficient quality for analysis. All surgical procedures were performed under general anesthesia; therefore, patients with ASA physical status I or II were included to ensure an acceptable perioperative risk profile and minimize the potential influence of significant systemic comorbidities on surgical outcomes. The exclusion criteria were as follows: (1) patients with tooth mobility grade II, III or IV; (2) a medical history of treatments or systemic diseases that may affect bone tissue metabolism (such as antiresorptive medications, prolonged corticosteroid therapy, active chemotherapy, head-and-neck radiotherapy, uncontrolled diabetes mellitus, osteoporosis); (3) a history of smoking, alcohol or drug use; (4) other conditions with the potential to impact the implant surgery procedure; and (5) incomplete or poor-quality radiographic records.

The patients were categorized into two groups: fully edentulous and partially edentulous maxilla. In the partially edentulous group, the requirement of four stable teeth, with a minimum of two teeth per quadrant, was established for the markerless tracing registration (MTR) protocol.

### 2.3. Surgical and Registration Protocol

Preoperative planning was based on CBCT scans. In the fully edentulous group, four bone-anchored mini-screws were placed in a polygonal configuration prior to CBCT imaging. The digital datasets were imported into the RealGUIDE software (version 5.4, 3DIEMME, Figino, Serenza, Italy), which was used for preoperative implant planning. All virtual implant planning was conducted by the same surgeon (I.D.). After virtual planning, the CBCT datasets were transferred to the dynamic navigation system.

All surgical procedures were performed under general anesthesia by the same surgeon with extensive clinical experience (I.D.). The patient tracker was attached to a head-mounted device positioned on the nasion and stabilized at the ears (head tracker). The registration process differed based on the patient’s edentulism. In partially edentulous patients, registration was achieved by “tracking” the incisal edges of anterior teeth or cusps of premolars or molars on four selected teeth. The four teeth were selected according to their availability and stability, with the aim of achieving a polygonal distribution across the arch and providing stable anatomical reference points for registration. In fully edentulous patients, due to the absence of teeth, registration was performed using four bone-anchored mini-screws. These screws were placed in a polygonal configuration to provide fixed fiducial markers for the optical tracing system and remained in position between CBCT imaging and surgery.

The tracking camera was positioned to detect the patient tracker and surgical instruments. Each navigated instrument was individually presented to the tracking camera, and its tip accuracy was verified. When necessary, instrument tip calibration was performed using the manufacturer-provided calibration block before proceeding with surgery. The calibration procedure established the relationship between the instrument tip and its optical tracking marker, enabling the navigation system to determine the position of the drill tip relative to the patient.

The registration probe was used to map reference points according to the patient‘s edentulism: distinct incisal edges or cusps on four selected teeth were traced in partially edentulous patients, whereas previously placed bone-anchored mini-screws were mapped in fully edentulous patients. The navigation system subsequently synchronized these physical reference points with the corresponding landmarks on the virtual 3D model derived from the preoperative CBCT dataset. Registration accuracy was verified before initiation of the osteotomy.

Following registration and accuracy verification, a full-thickness mucoperiosteal flap was raised. During implant site preparation, the surgical instruments were continuously tracked by the navigation system, and each surgical drill was individually calibrated before use. The navigation system provided real-time guidance from the initial position to the final planned trajectory and final position. After implant placement, a postoperative CBCT scan was acquired and imported into planning software for assessment of deviation between the virtually planned and clinically achieved implant positions.

### 2.4. Accuracy Assessment

**Figure 1:**
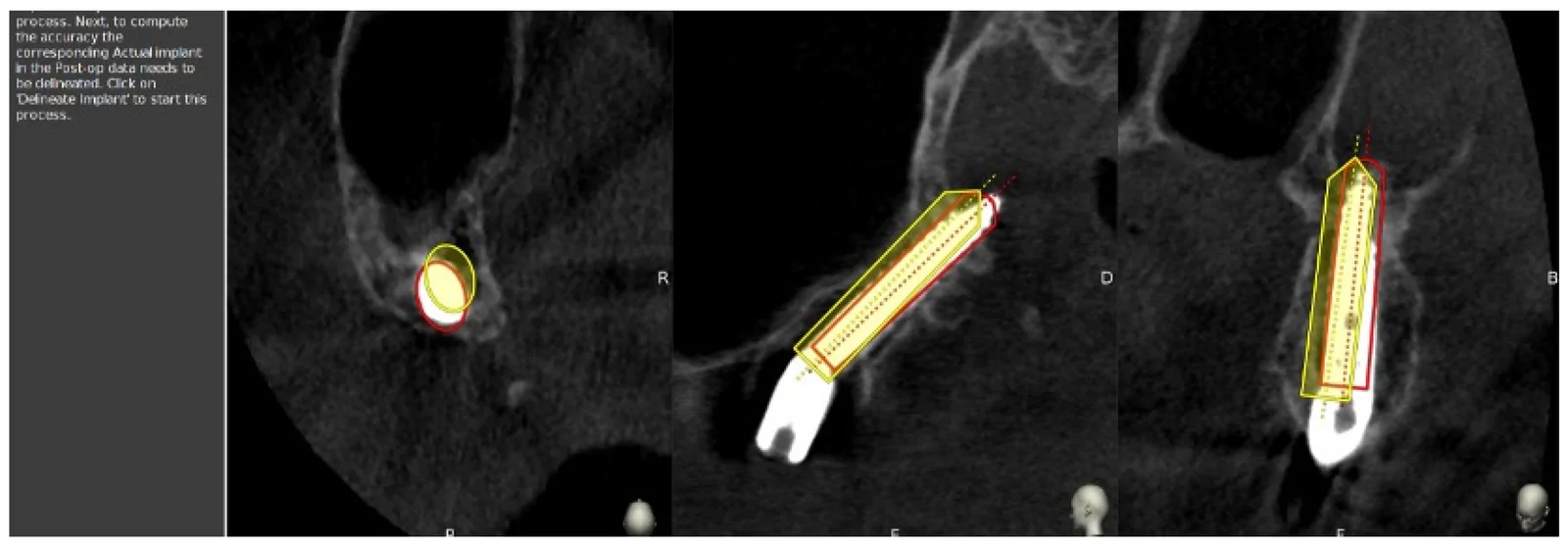
Accuracy assessment of pterygoid implants. Yellow, virtually planned implant; red, actual implant position.

Implant placement accuracy was evaluated by superimposing the virtually planned implant position with the actual implant position obtained from pre- and postoperative CBCT scans using the RealGUIDE software (3DIEMME, Italy). Preoperative and postoperative CBCT scans were obtained with the Planmeca ProMax (Planmeca, Helsinki, Finland) using the same parameters: 96 kV, 8 mA, 12 s, and 0.2 mm voxel size (Figure 1). The CBCT datasets were aligned using voxel-based registration based on stable anatomical structures unaffected by the surgical procedure: the cortical bone of the anterior maxillary plate, the nasal floor, and the zygomatic process. Following registration, the postoperative implant position was identified on the postoperative CBCT dataset and compared with the corresponding virtually planned implant position in RealGUIDE.

**Figure 2:**
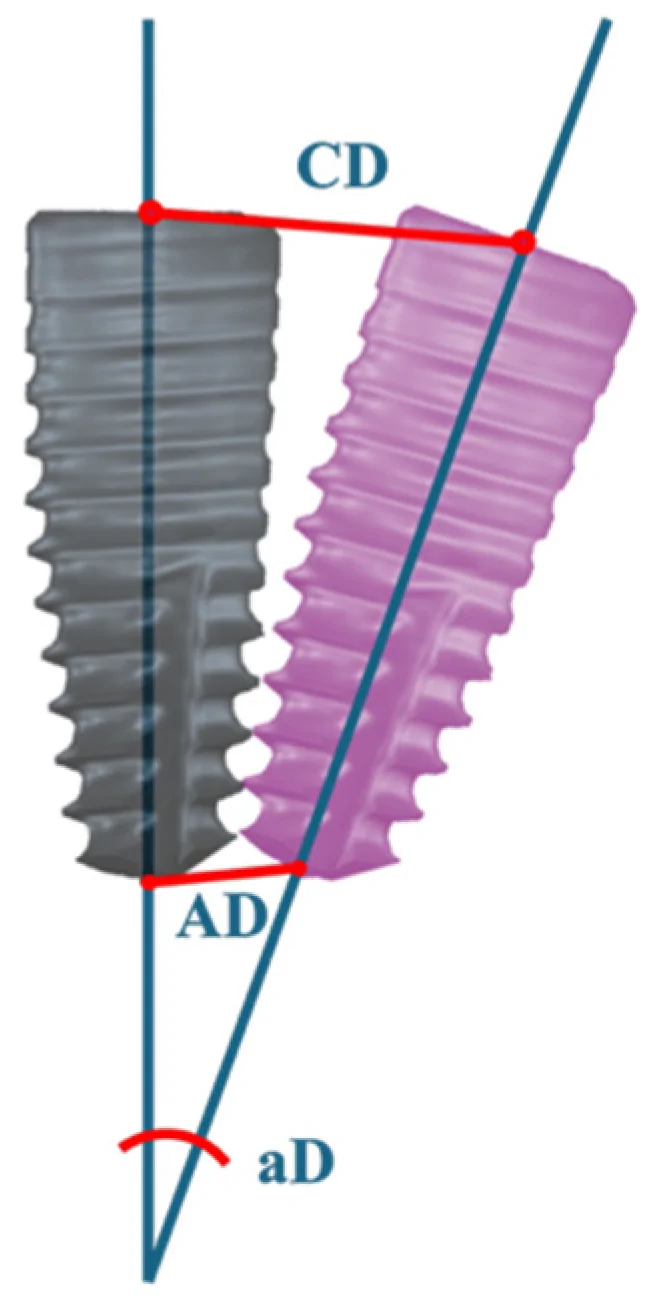
Evaluation of implant placement accuracy. Gray: virtually planned implant. Purple: actual implant position. CD—coronal deviation, AD—apical deviation, aD—angular deviation.

Three parameters were measured for each implant: coronal deviation (CD), apical deviation (AD), and angular deviation (aD) between the virtual plan and real position of the implant (Figure 2). Coronal and apical deviations were defined as the 3D global linear distances between the planned and clinically achieved implant positions at the midpoint of the implant shoulder and apex, respectively. Angular deviation was defined as the angle between the long center axes of the virtually planned and clinically achieved implant positions.

All image registration procedures and measurements were performed by a single experienced surgeon (I.D.). Each parameter was measured three times, and the arithmetic mean was recorded. As the measurements were performed by the treating surgeon, the possibility of observer-related measurement bias cannot be excluded.

### 2.5. Statistical Analyses

All analyses were performed using the Data Science Workbench, version 14 (Cloud Software Group, Inc., Fort Lauderdale, FL, USA, 2025). Continuous variables are presented as mean ± standard deviation (SD), median, range, and 95% confidence intervals (CI). Categorical variables are presented as frequencies and percentages. To verify the consistency of measurements, an intra-examiner reliability test was conducted. Ten randomly selected implants were re-evaluated by the same examiner after two weeks. The intra-class correlation coefficient (ICC) was 0.92, indicating excellent reliability and high internal consistency of the measurement protocol.

Normality testing was performed using the Shapiro–Wilk test, supplemented by visual inspection of Q-Q plots and box plots, and evaluation of skewness and kurtosis values. Coronal and apical deviations satisfied normality assumptions (Shapiro–Wilk *p* = 0.969 and *p* = 0.332, respectively; kurtosis within ±1.0) and were compared between groups using independent samples *t*-tests (two-tailed). The non-parametric Mann–Whitney test was used for angular deviation based on convergent evidence: a Shapiro–Wilk *p*-value (*p* = 0.054), a markedly platykurtic distribution (kurtosis = −1.21), and systematic tail deviations observed in QQ plot inspection. Effect sizes were calculated using Cohen’s d for parametric comparisons (coronal and apical deviation), with 95% CI estimated using the non-central t-distribution method. For angular deviation, rank-biserial correlation (r) was used as the effect size measure consistent with the non-parametric Mann–Whitney U test. Its 95% CI was estimated by converting the Cohen’s d confidence interval bounds using the formula r = d/√(d^2^ + 4). Effect sizes were classified as small, medium, and large with d values of 0.2, 0.5, and 0.8 and r values of 0.1, 0.3, and 0.5, respectively. Between-group differences are reported with 95% CI. The level of statistical significance was set at α = 0.05.

## 3. Results

A total of 40 pterygoid implants were placed in 20 fully edentulous patients and 20 partially edentulous patients. No statistically significant differences were observed in gender distribution, age, implant site, or length between groups. Table 1 summarizes the patient and implant characteristics stratified by study groups.

In fully edentulous patients, the mean deviation between the planned and actual implant position was 1.24 mm at the coronal level, 1.30 mm at the apical level, and 0.72° for angular deviation. In partially edentulous patients, the corresponding values were 1.40 mm, 1.30 mm, and 0.82°, respectively. No statistically significant differences were observed between the groups. However, partially edentulous patients showed slightly higher mean deviations at the coronal level and in angular measurements. The results are summarized in Table 2.

Effect size analysis provided additional context beyond *p*-values. Coronal deviation demonstrated a medium effect size (d = 0.60, 95% CI: 0.07 to 1.13). Apical deviation showed a negligible effect (d = 0.002, 95% CI: −0.64 to 0.64), while angular deviation demonstrated a small effect (r = 0.11, 95% CI: −0.42 to 0.86).

## 4. Discussion

The primary objective of this retrospective study was to evaluate the accuracy of pterygoid implant placement using the dCAIS system in fully and partially edentulous maxillae. Accuracy was defined as the 3D deviation between the actual implant position obtained from the postoperative CBCT scan and the virtually planned implant position established during the preoperative phase.

The results of this study suggest that the placement of pterygoid implants using dCAIS may provide predictable accuracy regardless of the degree of edentulism. No statistically significant differences were observed in coronal, apical, and angular deviations of pterygoid implants between the fully and partially edentulous groups. Importantly, the registration method differed between the two groups. Bone-anchored mini-screws were used in fully edentulous patients, whereas tooth-based trace registration was used in partially edentulous patients. Therefore, the lack of statistically significant differences between the two groups may be influenced by several group-specific factors rather than the registration method itself, including anatomical access, residual dentition, intraoperative visibility, drill interference, and the distribution and position of fiducial markers.

The distribution of fiducial markers may also play an important role in registration accuracy. West et al. reported that registration precision is maximized when fiducial markers are widely spread in a non-linear (polygonal) arrangement and when the centroid of the set of markers is positioned closer to the operative region [38]. For pterygoid implants, placing fiducial markers on the maxillary tuberosity may help reduce target registration error (TRE) in dCAIS [39]. However, this potential influence could not be isolated in the present study because registration technique and edentulism status were intrinsically linked.

To date, few studies have analyzed pterygoid implant placement with dynamic navigation. Stefanelli et al. reported a mean deviation of 0.66 mm at the coronal level, 1.13 mm at the apical level, and 2.64° of angular deviation between planned and actual position using the MTR method [40]. Similarly, Tao et al. observed mean deviations of 0.93 mm at the coronal level, 1.37 mm at the apical level, and an angular deviation of 2.41° in vitro at fully edentulous maxillae using the RMR method [41]. In comparison, the present study demonstrated a mean deviation for fully and partially edentulous maxillae of 1.24 ± 0.27 mm and 1.40 ± 0.27 mm at the coronal level, 1.30 ± 0.49 mm and 1.30 ± 0.54 at the apical level, and 0.72 ± 0.38° and 0.82 ± 0.43° in angular deviation, respectively. No statistically significant between-group differences were detected for these parameters. The angular deviation observed in this study was lower than previously reported values. This may be related to several factors, including the rigid stabilization provided by the head tracker, reduced patient movement under general anesthesia, technologically advanced computing in recent years, and surgeons’ experience. However, these explanations remain speculative as the present study was not designed to assess their individual effects. Further prospective studies with controlled conditions are needed to determine whether these factors contribute to improved angular accuracy.

While coronal deviation was slightly higher, apical deviation remained consistent with prior findings. The discrepancy in coronal accuracy may be related to the number of landmarks utilized during the registration process. Stefanelli et al. demonstrated that tracing 5–6 landmarks (teeth) resulted in significantly higher precision compared to tracing only 3–4 points during the registration process [37]. In the fully edentulous maxilla, Fun et al. demonstrated that with the use of at least five fiducial markers can achieve optimal TRE values with high accuracy in zygomatic implant placement [42]. In the present study, four fiducial markers were used for registration; therefore, increasing the number of landmarks might minimize the initial entry shift. Further investigation is required to determine whether increasing the number of landmarks improves registration accuracy.

Another objective of this study was to assess whether the presence of teeth affects dCAIS accuracy. In partially edentulous patients, the presence of stable teeth provides reliable fiducial markers for the trace registration (TR) protocol. However, these patients exhibited slightly higher mean deviation in coronal (1.40 mm vs. 1.24 mm) and angular (0.82° vs. 0.72°) parameters compared to fully edentulous patients. This difference could be explained by the mechanical interference of remaining teeth with handpiece range of motion and reduced visibility of long pterygoid drills, in contrast to the “open-field” of fully edentulous patients. However, other differences between the groups may have also contributed to these findings.

An in vitro study comparing the effects of RMR and MTR on implant placement using dCAIS showed significant differences in mean deviation at the coronal level (1.53 mm vs. 0.83 mm) and the apical level (1.63 mm vs. 1.07 mm), respectively [35]. In that study, the RMR method utilized a thermoplastic intraoral clip with a radiographic marker adapted to the remaining teeth, while the MTR method utilized an optical probe to trace four fiducial points on cusps or edges of the teeth specifically selected to form the largest possible geometric distribution. Although the authors concluded that MTR seems to increase the accuracy of implant placement, the difference in angular deviation (4.30° vs. 3.89°) was not found to be statistically significant [35]. In the present study, bone-anchored mini-screws were used for registration in fully edentulous patients, which may provide more rigid fixation than the thermoplastic clips and potentially reduce marker displacement.

In comparison with the deviations reported for free-hand (FH) placement of pterygoid implants, which often exceeded 10° in angular deviation, 1.5 mm at the coronal level, and 2.7 mm at the apical level, the deviations observed in the present study were generally lower [40,41]. Previous studies have reported improved accuracy with static CAIS compared with the FH approach [40,41]. A systematic review and meta-analysis of sCAIS accuracy reported a mean angular deviation of 3.59°, a coronal deviation of 1.02 mm, and an apical deviation of 1.33 mm [43]. These values are consistent with the 2018 ITI consensus report, which concluded that a mean coronal deviation of 1.2 mm, an apical deviation of 1.5 mm, and an angular deviation of 3.5° are clinically acceptable and safe in most cases [44]. In contrast, our results demonstrated a lower mean angular deviation of 0.76°. This suggests that dynamic navigation can achieve good angular accuracy in pterygoid implant placement [45]. Further studies under similar clinical conditions are needed to determine whether either approach offers an advantage in angular accuracy. Future developments in dCAIS may include the integration of augmented reality-assisted surgery, which could provide real-time visualization of planned implant positions and relevant anatomic structures during surgery [46].

The present study has several limitations that should be noted. First, its retrospective design of the study may be subject to selection bias and limits the ability to control potentially important confounding variables. Second, the relatively small sample size could lack the statistical power to detect between-group differences. No sample-size calculation was performed, and the findings should be considered exploratory. Larger prospective, multicenter studies are required to confirm these results. Furthermore, all surgical procedures were performed by a single, experienced surgeon, which ensured consistency, eliminating inter-operator variability; however, this may limit the external validity of the findings when applied to clinicians with less experience [47]. Also, the measurements were performed by the treating surgeon, which may introduce measurement bias. A further important limitation is the confounding between edentulism status and registration technique. The fully edentulous group received RMR with bone-anchored mini-screws, whereas the partially edentulous group received tooth-based MTR. Consequently, the present study cannot determine whether any observed difference in accuracy was attributable to edentulism, registration technique, or other associated anatomical and procedural characteristics. The findings should therefore be regarded as supportive evidence of the accuracy achievable with dCAIS rather than evidence of equivalence or superiority between registration protocols. Although our findings demonstrate the accuracy achievable with dCAIS, the lack of a freehand control group prevents a direct comparison with conventional implant placement, limiting our ability to assess the added benefit of dCAIS. Additionally, all surgeries were carried out under general anesthesia, reducing patients’ movement and potentially improving accuracy; therefore, the results might differ if the interventions were performed under local anesthesia.

## 5. Conclusions

No statistically significant between-group differences in placement accuracy were detected in coronal, apical, and angular deviations between the bone-anchored radiographic marker registration (RMR) workflow used in fully edentulous patients and the tooth-supported markerless tracing registration (MTR) workflow used in partially edentulous patients. These findings should be interpreted cautiously because of the limited sample size, retrospective study design, single-operator setting, and the intrinsic association between registration method and edentulism status.

## Figures and Tables

**Table 1:** Patient and implant characteristics stratified by study group.

Characteristic	Total (N = 40)	Edentulous (n = 20)	Partially Edentulous (n = 20)	*p*-Value
**Gender, n (%)**				0.751 †
Female	21 (52.5%)	10 (50.0%)	11 (55.0%)	
Male	19 (47.5%)	10 (50.0%)	9 (45.0%)	
**Age (years)**				0.081 ‡
Mean ± SD	55.1 ± 6.6	53.3 ± 6.9	56.9 ± 5.9	
Median	56.0	54.0	56.0	
Range	40–69	40–67	47–69	
**Side, n (%)**				0.058 †
Right	20 (50.0%)	7 (35.0%)	13 (65.0%)	
Left	20 (50.0%)	13 (65.0%)	7 (35.0%)	
**Implant Length, n (%)**				
18 mm	33 (82.5%)	16 (80.0%)	17 (85.0%)	0.709 †
20 mm	7 (17.5%)	4 (20.0%)	3 (15.0%)	

† Chi-square test or Fisher’s exact test. ‡ Independent samples *t*-test.

**Table 2:** Differences between the two study groups (fully edentulous and partially edentulous) for all accuracy variables.

Parameter	Edentulous (n = 20)	Partially Edentulous (n = 20)	Mean Diff. (95% CI)	Relative Diff.	*p*-Value
**Coronal Deviation (mm)**					
Mean ± SD	1.24 ± 0.27	1.40 ± 0.27	+0.16 (−0.01 to 0.33)	+12.9%	0.065 †
Median	1.22	1.42	-	-	-
95% CI	1.12–1.36	1.27–1.53	-	-	-
Range	0.64–1.70	0.98–1.98	-	-	-
**Apical Deviation (mm)**					
Mean ± SD	1.30 ± 0.49	1.30 ± 0.54	+0.001 (−0.33 to 0.33)	0.0%	0.995 †
Median	1.31	1.20	-	-	-
95% CI	1.07–1.53	1.04–1.55	-	-	-
Range	0.34–2.14	0.39–2.20	-	-	-
**Angular Deviation (°)**					
Mean ± SD	0.72 ± 0.38	0.82 ± 0.43	+0.10 (−0.16 to 0.36)	+13.9%	0.560 ‡
Median	0.77	0.76	-	-	-
95% CI	0.54–0.90	0.62–1.02	-	-	-
Range	0.12–1.22	0.28–2.24	-	-	-

† Independent samples *t*-test (two-tailed). ‡ Mann–Whitney U test (two-tailed) applied due to non-normal distribution of angular deviation (Shapiro–Wilk *p* = 0.054, kurtosis = −1.21). The 95% CI for mean differences was calculated using the pooled standard error; for angular deviation, the 95% CI represents a normal approximation of the difference in means.

## Data Availability

The data presented in this study are available from the corresponding author upon reasonable request. The data are not publicly available due to privacy and ethical restrictions.

## Abbreviations

The following abbreviations are used in this manuscript:

3D	Three-dimensional
AD	Apical deviation
aD	Angular deviation
CAIS	Computer-aided implant surgery
CBCT	Cone-beam computed tomography
CD	Coronal deviation
CI	Confidence interval
dCAIS	Dynamic computer-assisted implant surgery system
ICC	Intraclass correlation coefficient
MTR	Markerless tracing registration
RMR	Radiographic marker registration
sCAIS	Static computer-aided implant surgery
SD	Standard deviation
TRE	Target registration error
